# *Veillonella*, *Firmicutes*: Microbes disguised as Gram negatives

**DOI:** 10.4056/sigs.2981345

**Published:** 2013-12-15

**Authors:** Tammi Vesth, Aslı Ozen, Sandra C. Andersen, Rolf Sommer Kaas, Oksana Lukjancenko, Jon Bohlin, Intawat Nookaew, Trudy M. Wassenaar, David W. Ussery

**Affiliations:** 1 Center for Biological Sequence Analysis, Department of Systems Biology, Technical University of Denmark, Lyngby, Denmark; 2 Norwegian School of Veterinary Science, Department of Food Safety and Infection Biology, Oslo, Norway; 3 Department of Chemical and Biological Engineering, Chalmers University of Technology, Gothenburg, Sweden; 4 Molecular Microbiology and Genomics Consultants, Zotzenheim, Germany; 5 The Novo Nordisk Foundation Center for Biosustainability, Technical University of Denmark; 6 Comparative Genomics Group, Biosciences Division, Oak Ridge National Laboratory, Oak Ridge, Tennessee 37831, USA

## Abstract

The *Firmicutes* represent a major component of the intestinal microflora. The intestinal *Firmicutes* are a large, diverse group of organisms, many of which are poorly characterized due to their anaerobic growth requirements. Although most *Firmicutes* are Gram positive, members of the class *Negativicutes*, including the genus *Veillonella*, stain Gram negative. *Veillonella* are among the most abundant organisms of the oral and intestinal microflora of animals and humans, in spite of being strict anaerobes. In this work, the genomes of 24 *Negativicutes*, including eight *Veillonella* spp., are compared to 20 other *Firmicutes* genomes; a further 101 prokaryotic genomes were included, covering 26 phyla. Thus a total of 145 prokaryotic genomes were analyzed by various methods to investigate the apparent conflict of the *Veillonella* Gram stain and their taxonomic position within the *Firmicutes*. Comparison of the genome sequences confirms that the *Negativicutes* are distantly related to *Clostridium* spp., based on 16S rRNA, complete genomic DNA sequences, and a consensus tree based on conserved proteins. The genus *Veillonella* is relatively homogeneous: inter-genus pair-wise comparison identifies at least 1,350 shared proteins, although less than half of these are found in any given *Clostridium* genome. Only 27 proteins are found conserved in all analyzed prokaryote genomes. *Veillonella* has distinct metabolic properties, and significant similarities to genomes of *Proteobacteria* are not detected, with the exception of a shared LPS biosynthesis pathway. The clade within the class *Negativicutes* to which the genus *Veillonella* belongs exhibits unique properties, most of which are in common with Gram-positives and some with Gram negatives. They are only distantly related to *Clostridia*, but are even less closely related to Gram-negative species. Though the *Negativicutes* stain Gram-negative and possess two membranes, the genome and proteome analysis presented here confirm their place within the (mainly) Gram positive phylum of the *Firmicutes*. Further studies are required to unveil the evolutionary history of the *Veillonella* and other *Negativicutes*.

## Background

The genus *Veillonella,* belonging to *Negativicutes,* consists of anaerobic, non-fermentative, Gram-negative cocci, that are normally observed in pairs or short chains, and are non-sporulating and non-motile [1]. *Veillonella* spp. are abundant in the human microbiome and are found in the oral, respiratory, intestinal and genitourinary flora of humans and animals; they can make up as much as 10% of the bacterial community initially colonizing the enamel [2] and are found throughout the entire oral cavity [3], especially on the tongue dorsum and in saliva [4]. The importance of *Veillonella* spp. in human infections is uncertain, and they are generally considered to be of low virulence. *Veillonella* form biofilms, often with *Streptococcus* spp., and species of these genera have been found to be more abundant in the oral microflora of people with poor oral health [5]. Studies have shown that during formation of early dental plaque, the fraction of *Veillonella* spp. changes in mixed-microbial colonies with streptococci [6]. Thus, *Veillonella* spp. may play a role in caries formation as they utilize the lactic acid produced by the organisms conducive to caries [7]. *Veillonella* are also among the most common anaerobic species reported from pulmonary samples and are frequently recovered from cystic fibrosis cases [8]. The organisms are also abundant in the human gut flora, where their numbers were found to be higher in children with type I diabetes compared to healthy controls [9]. Currently, 12 species of *Veillonella* have been characterized [10,11] including *V. parvula*, *V. atypica* and *V. dispar*, which are found in the human oral cavity.

The *Negativicutes* are the only diderm (literally 'two skins') members of the phylum *Firmicutes* as they possess an inner and an outer membrane. Their placement within the *Firmicutes* has been widely accepted, and has been confirmed by 16S rRNA analysis [12]. However, their genomes have not been analyzed in detail to confirm their taxonomic position. This work presents a broad analysis of the *Negativicutes* with focus on the *Veillonella* spp. using comparative microbial genomics. A total of 24 genomes from the *Negativicutes* were compared to 121 genomes covering most of the taxonomic span of sequenced bacterial genomes. We investigated how the *Negativicutes* genomes compared to other bacterial genomes using three different and complementary approaches: 1) phylogenetic trees to visualize the relative distance of the *Negativicutes* genomes to other genomes; 2) amino acid composition, nucleotide tetramer frequency and metabolism analysis using 2-D clustering and heatmaps to compare genomes; and 3) proteomic comparison across the *Negativicutes* genomes.

## Materials and Methods

### Genome sequences used for analysis

The set of 145 genomes included in this study (24 *Negativicutes* genomes and 121 other prokaryotic genomes covering 26 phyla) are listed in Table 1.

**Table 1:** Genomes used in this study

Phylum	Name of organism and strain	Strain designation	Type strain?	NCBI Taxon ID	NCBI Project ID
*Acidobacteria*	*Acidobacterium capsulatum *	ATCC 51196	Yes	240015	28085
*Acidobacteria*	*“Korebacter versatiles” *	Ellin 345		204669	15771
*Acidobacteria*	*“Solibacter usitatus” *	Ellin6076		234267	12638
*Actinobacteria*	*Bifidobacterium bifidum *	317B	No	1681	42863
*Actinobacteria*	*Catenulispora acidiphila *	ID139908, DSM 44928	Yes	479433	21085
*Actinobacteria*	*Corynebacterium pseudotuberculosis *	C231	No	681645	40875
*Actinobacteria*	*Segniliparus rugosus *	ATCC BAA-974	Yes	679197	40685
*Actinobacteria*	*Streptomyces bingchenggensis *	BCW-1	Name not validly published	749414	46847
*Actinobacteria*	*Tropheryma whipplei *	Twist	Yes	*203267*	*95*
*Aquificae*	*Persephonella marina *	EX-H1	Yes	123214	12526
*Aquificae*	*Sulfurihydrogenibium sp. *	YO3AOP1	No type strain available	436114	18889
*Aquificae*	*Thermocrinis albus *	HI 11/12, DSM 14484	Yes	638303	37275
*Bacteroidetes*	*Bacteroides thetaiotaomicron *	VPI-5482	Yes	226186	399
*Bacteroidetes*	*Candidatus* Sulcia muelleri	DMIN		641892	37785
*Bacteroidetes*	*Chitinophaga pinensis *	UQM 2034, DSM 2588	Yes	485918	27951
*Bacteroidetes*	*Paludibacter propionicigenes *	WB4, DSM 17365	Yes	694427	42009
*Chlamydiae*	*Protochlamydia amoebophila *	UWE25	Yes	264201	10700
*Chlamydiae*	*Chlamydia trachomatis *	E/Sweden2	No	634464	43167
*Chlamydiae*	*Chlamydophila pneumoniae *	AR39	No	115711	247
*Chlamydiae*	*Waddlia chondrophila *	WSU 86-1044	Yes	716544	43761
*Chlorobi*	*“Chlorobium chlorochromatii” *	CaD3	Name not validly published	340177	13921
*Chlorobi*	*Chlorobium tepidum *	TLS	Yes	194439	302
*Chloroflexi*	*Chloroflexus aggregans *	DSM 9485	Yes	326427	16708
*Chloroflexi*	*Dehalococcoides sp *	BAV1	No	216389	15770
*Chloroflexi*	*Herpetosiphon aurantiacus *	ATCC 23779	Yes	316274	16523
*Chloroflexi*	*Roseiflexus *sp.	RS-1	No type strain available	357808	16190
*Cyanobacteria*	*Anabaena variabilis* 3	ATCC 2941	No	240292	10642
*Cyanobacteria*	*Cyanothece sp. *	PCC 7822	No	497965	28535
*Cyanobacteria*	*Prochlorococcus marinus *	MIT9301	No	167546	15746
*Cyanobacteria*	*Synechocystis sp. *	PCC6803	No	1148	60
*Deferribacteres*	*Calditerrivibrio nitroreducens *	Yu37-1, DSM 19672	Yes	768670	49523
*Deferribacteres*	*Deferribacter desulfuricans *	SSM1, DSM 14783	Yes	197162	37285
*Deferribacteres*	*Denitrovibrio acetiphilus *	N2460, DSM 12809	Yes	522772	29431
*Deinococcus-Thermus*	*Oceanithermus profundus *	506, DSM 14977	Yes	670487	40223
*Deinococcus-Thermus*	*Thermus thermophilus *	HB8	Yes	300852	13202
*Deinococcus-Thermus*	*Truepera radiovictrix *	RQ-24, DSM 17093	Yes	649638	38371
*Dictyoglomi*	*Dictyoglomus turgidum *	DSM 6724	Yes	515635	29175
*Elusimicrobia*	*Elusimicrobium minutum *	Pei 191	Yes	445932	19701
*Fibrobacteres*	*Fibrobacter succinogenes *	S85	Yes	59374	32617
*Firmicutes*	*Acetohalobium arabaticum *	Z-7288, DSM 5501	Yes	574087	32769
*Firmicutes*	*Acidaminococcus fermentans *	VR4, DSM 20731	Yes	591001	33685
*Firmicutes*	*Acidaminococcus sp. *	D21	No type strain available	563191	34117
*Firmicutes*	*Alkaliphilus oremlandii *	OhILAs	Yes	350688	16083
*Firmicutes*	*Bacillus subtilis* subsp. *subtilis *	168	Yes	224308	76
*Firmicutes*	*Clostridium botulinum *	F Langeland	No	441772	19519
*Firmicutes*	*Clostridium cellulolyticum *	H10	Yes	394503	17419
*Firmicutes*	*Clostridium difficile *	630 (epidemic type X)	No	272563	78
*Firmicutes*	*“Desulfotomaculum reducens” *	MI-1	Name not validly published	349161	13424
*Firmicutes*	*Dialister invisus *	DSM 15470	Yes	592028	33143
*Firmicutes*	*Dialister micraerophilus *	Oral Taxon 843 DSM 19965	Yes	888062	53029
*Firmicutes*	*Dialister micraerophilus *	UPII-345-E	No	910314	59521
*Firmicutes*	*Enterococcus faecalis *	V583	No	226185	70
*Firmicutes*	*Eubacterium cylindroides *	T2-87	No	717960	45917
*Firmicutes*	*Eubacterium rectale *	A1-86, DSM 17629	No	39491	39159
*Firmicutes*	*Exiguobacterium sibiricum *	255-15	Yes	262543	10649
*Firmicutes*	*Geobacillus kaustophilus *	HTA426	Yes	235909	13233
*Firmicutes*	*Lactococcus lactis *	cremoris MG1363	No	416870	18797
*Firmicutes*	*Lysinibacillus sphaericus *	C3-41	No	444177	19619
*Firmicutes*	*Megamonas hypermegale *	ART12/1	No	158847	39163
*Firmicutes*	*Megasphaera genomo *sp.	type 1 28L	No type strain available	699218	42553
*Firmicutes*	*Megasphaera micronuciformis *	F0359	No	706434	43125
*Firmicutes*	*Mitsuokella multacida *	A 405-1, DSM 20544	Yes	500635	28653
*Firmicutes*	*Paenibacillus sp. *	JDR-2	No	324057	20399
*Firmicutes*	*Phascolarctobacterium sp. *	YIT 12067	No	626939	48505
*Firmicutes*	*Selenomonas artemidis *	F0399	No	749551	47277
*Firmicutes*	*Selenomonas flueggei *	ATCC 43531	Yes	638302	37273
*Firmicutes*	*Selenomonas noxia *	ATCC 43541	Yes	585503	34641
*Firmicutes*	*Selenomonas *sp.	Oral Taxon 137 F0430	No type strain available	879310	52055
*Firmicutes*	*Selenomonas *sp.	Oral Taxon 149 67H29BP	No type strain available	864563	50535
*Firmicutes*	*Selenomonas sputigena *	DSM 20758	Yes	546271	51247
*Firmicutes*	*Staphylococcus aureus aureus*	ED98	No	681288	39547
*Firmicutes*	*Streptococcus pneumoniae *	TIGR4	No	170187	277
*Firmicutes*	*Thermoanaerobacter *sp.	X514	Name not validly published	399726	16394
*Firmicutes*	*Thermosinus carboxydivorans *	Nor1	Yes	401526	17587
*Firmicutes*	*Turicibacter *sp.	PC909 702450 42765	No		
*Firmicutes*	*Veillonella atypica *	ACS-049-V-Sch6	No	866776	51075
*Firmicutes*	*Veillonella atypica *	ACS-134-V-Col7a	No	866778	51079
*Firmicutes*	*Veillonella dispar *	ATCC 17748	Yes	546273	30491
*Firmicutes*	*Veillonella parvula *	ATCC 17745	No	686660	41557
*Firmicutes*	*Veillonella parvula *	Te3, DSM 2008	Yes	479436	21091
*Firmicutes*	*Veillonella sp. *	3 1 44	Name not validly published	457416	41975
*Firmicutes*	*Veillonella sp. *	6 1 27	Name not validly published	450749	41977
*Firmicutes*	*Veillonella sp. *	Oral Taxon 158 F0412	Name not validly published	879309	52053
*Fusobacteria*	*Fusobacterium nucleatum nucleatum*	ATCC 25586	Yes	190304	295
*Fusobacteria*	*Ilyobacter polytropus *	CuHBu1, DSM 2926	Yes	572544	32577
*Fusobacteria*	*Leptotrichia buccalis *	C-1013-b, DSM 1135	Yes	523794	29445
*Fusobacteria*	*Sebaldella termitidis *	NCTC 11300	Yes	526218	29539
*Fusobacteria*	*Streptobacillus moniliformis *	9901, DSM 12112	Yes	519441	29309
*Planctomycetes*	*Pirellula staleyi *	DSM 6068	Yes	530564	29845
*Planctomycetes*	*Planctomyces limnophilus *	Mu 290, DSM 3776	Yes	521674	29411
*Proteobacteria*	*Acinetobacter baumannii *	SDF	No	509170	13001
*Proteobacteria*	*Alkalilimnicola ehrlichii *	MLHE-1	Yes	187272	15763
*Proteobacteria*	*Arcobacter nitrofigilis *	DSM 7299	Yes	572480	32593
*Proteobacteria*	*Burkholderia xenovorans *	(fungorum) LB400	Yes	266265	254
*Proteobacteria*	*Campylobacter jejuni *	doylei 269.97	No	360109	17163
*Proteobacteria*	*Candidatus* *Pelagibacter ubique*	SAR11 HTCC1062	Name not validly published	335992	13989
*Proteobacteria*	*Candidatus Zinderia insecticola*	CARI	Name not validly published	871271	51243
*Proteobacteria*	*Cellvibrio japonicus *	Ueda107	Yes	498211	28329
*Proteobacteria*	*Cupriavidus taiwanensis *	LMG19424	Yes	164546	15733
*Proteobacteria*	*Escherichia coli *	K-12, MG1655	No	511145	225
*Proteobacteria*	*Geobacter uraniireducens *	Rf4	Yes	351605	15768
*Proteobacteria*	*Hahella chejuensis *	KCTC 2396	Yes	349521	16064
*Proteobacteria*	*Haliangium ochraceum *	SMP-2, DSM 14365	Yes	502025	28711
*Proteobacteria*	*Helicobacter pylori *	908	No	869727	50869
*Proteobacteria*	*Lawsonia intracellularis *	PHE/MN1-00	No	363253	183
*Proteobacteria*	*Magnetococcus *sp.	MC-1	Name not validly published	156889	262
*Proteobacteria*	*Methylobacterium nodulans *	ORS2060	Yes	460265	20477
*Proteobacteria*	*Neisseria meningitidis *	Z2491	No	122587	252
*Proteobacteria*	*Neorickettsia sennetsu *	Miyayama	Yes	222891	357
*Proteobacteria*	*Nitrosomonas eutropha *	C91 (C71)	Yes	335283	13913
*Proteobacteria*	*Photorhabdus luminescens laumondii *	TT01	Yes	243265	9605
*Proteobacteria*	*Polynucleobacter necessarius *	STIR1	No	452638	19991
*Proteobacteria*	*Pseudomonas aeruginosa*	LESB58	No	557722	31101
*Proteobacteria*	*Pseudomonas fluorescens*	SBW25	No	216595	31229
*Proteobacteria*	*Pseudomonas stutzeri*	A1501	No	379731	16817
*Proteobacteria*	*Salmonella enterica enterica*	PT4 P125109	No	550537	30687
*Proteobacteria*	*Shewanella oneidensis *	MR-1	Yes	211586	335
*Proteobacteria*	*Sorangium cellulosum *	So ce56	No	448385	28111
*Proteobacteria*	*Stigmatella aurantiaca *	DW4 /3-1	No	378806	52561
*Proteobacteria*	*Sulfurospirillum deleyianum *	5175, DSM 6946	No	525898	29529
*Proteobacteria*	*Vibrio cholerae *	O395	No	345073	32853
*Spirochaetes*	*Borrelia turicatae *	91E135	Yes	314724	13597
*Spirochaetes*	*Brachyspira murdochii *	56-150, DSM 12563	Yes	526224	29543
*Spirochaetes*	*Leptospira interrogans *	lai 56601	No	189518	293
*Synergistetes*	*Thermanaerovibrio acidaminovorans *	Su883, DSM 6589	Yes	525903	29531
*Tenericutes*	*Acholeplasma laidlawii *	PG-8A	No	441768	19259
*Tenericutes*	*Candidatus* Phytoplasma asteris	yellows witches'-broom AY-WB 322098	Name not validly published	13478	
*Tenericutes*	*Candidatus* Phytoplasma mali	AT	Name not validly published	37692	25335
*Tenericutes*	*Mycoplasma genitalium *	G37	Yes	243273	97
*Tenericutes*	*Mycoplasma pneumoniae *	FH	No	722438	49525
*Tenericutes*	*Ureaplasma parvum *	sv 3, ATCC 27815	No	505682	19087
*Thermotogae*	*Fervidobacterium nodosum *	Rt17-B1	Yes	381764	16719
*Thermotogae*	*Kosmotoga olearia *	TBF 19.5.1	Yes	521045	29419
*Thermotogae*	*Petrotoga mobilis *	SJ95	Yes	403833	17679
*Thermotogae*	*Thermotoga naphthophila *	RKU-10	Yes	590168	33663
*Verrucomicrobia*	*Akkermansia muciniphila *	ATCC BAA-835	Yes	349741	20089
*Verrucomicrobia*	*Opitutus terrae*		Yes	PB90-1	452637
*Crenarchaeota*	*Sulfolobus solfataricus *	P2		273057	108
*Crenarchaeota*	*Thermosphaera aggregans *	M11TL, DSM 11486	Yes	633148	36571
*Euryarchaeota*	*Halogeometricum borinquense *	PR3, DSM 11551	Yes	469382	20743
*Euryarchaeota*	*Methanocella *sp.	RC-I	Name not validly published	351160	19641
*Euryarchaeota*	*Methanothermus fervidus *	V24S, DSM 2088	Yes	523846	33689
*Korarchaeota*	*Candidatus* Korarchaeum cryptofilum	OPF8	Name not validly published	374847	16525
*Nanoarchaeota*	*“Nanoarchaeum equitans” *	Kin4-M	Name not validly published	228908	9599

### 16S rRNA tree

For this analysis, 16S rRNA sequences were predicted from the whole genome sequences of the selected organisms, using the RNAmmer algorithm [13]. These sequences were aligned using the MAFFT program, with the iterative refinement algorithm using maximum iteration (1000) and default parameters for gap penalties [14]. A distance tree was constructed using MEGA5 [15] with the Neighbor-joining algorithm [16] and 1,000 bootstrap re-samplings. The taxa in the resulting tree were collapsed to phyla, except for the *Negativicutes*.

### Composition Vector Tree (CV)

A Composition Vector Tree was constructed based on protein sequences of the 145 selected genomes using a webserver (available at http://tlife.fudan.edu.cn/cvtree/) with the K parameter set at 6 [17]. The outcome from the program is a distance matrix based on amino acid sequence comparisons, which is then used to generate a phylogenetic tree with the neighbor-joining method. In the shown tree, the outgroup chosen was *Methanothermus fervidus* (an *Archaea*). After tree visualization with MEGA5, branches were collapsed wherever possible with the exception of the *Negativicutes* branch, which remained expanded.

### Consensus tree of conserved genes

Using the list of universally conserved core genes, previously identified by Ciccarelli *et al.* [18], and an implementation of BLAST, a set of genes that was shared among all 145 genomes was identified. Proteins that had no match in at least one genome or showed poor E-value were eliminated. The 27 conserved core genes were extracted (Table 1) and a multiple alignment was produced using MUSCLE software [19]. A set of phylogenetic trees was constructed by PAUP [20] and a best-fit consensus tree was generated using Phylogeny Inference package (PHYLIP) as described elsewhere [21]. Bootstrap values were found after 27 re-samplings, which is equal to the number of gene families conserved in all the analyzed genomes.

### DNA tetramer analysis and amino acid usage

A tetramer frequency heatmap was constructed from the observed ratios of tetra-nucleotide frequencies divided by estimated tetra-nucleotide frequencies for each genome [22]. The estimated tetra-nucleotides were computed from the genomes' base composition. The ratio of observed over expected frequency was used for hierarchical clustering using complete linkage and Euclidean distance, which was subsequently performed with respect to both strain and tetramer frequencies.

The amino acid heatmap is based on frequencies of deduced proteomic amino acids from each genome normalized with respect to the total number of amino acids in each genome. The amino acid frequencies for each genome were clustered using complete linkage and Euclidean distance with respect to both genomes and amino acids. The heatmap was made using the R package ggplot2 [23].

### Comparison of metabolism potential

The protein sequences of Kyoto Encyclopedia of Genes and Genomes (KEGG) orthology categories [24] were downloaded and only the Bacterial sequences were considered. The Hidden Markov model (HMM) of each ortholog was generated using HMMER version 3 [25] based on the multiple alignment of each orthologous set of KEGG proteins, using MUSCLE software [19]. The 145 proteomes were queried against the HMMs to infer their ontology. A cutoff of 1×10^−30^ was used for statistical significance. A heatmap of each pathway and process derived from the database KEGG was illustrated based on normalized abundance of the enzymes present in each pathway. The heatmap and hierarchical clustering were performed in the software R [23].

### Construction of BLAST matrix and proteome comparison

Reciprocal BLAST was performed between each genome pair. The program blastall version 2.2.25 was used for BLAST implementation using default settings (BLASTp, E-value set to 1×10^−5^ for non-homologs and 1×10^−8^ for homologs, without filtering). A hit was considered significant at a BLAST cutoff of 95% identity and 95% coverage (of the longest gene in comparison). The number of hits was then given as a percentage of the genes in the column representing the corresponding genome. The diagonal designates internal homologs, computed by blasting each genome with itself. To avoid including identical genes, the second highest scoring hits were used. Furthermore, we also performed homology reduction of the diagonal hits, using an implementation of the Hobohm algorithm [26].

## Results

**Figure 1:**
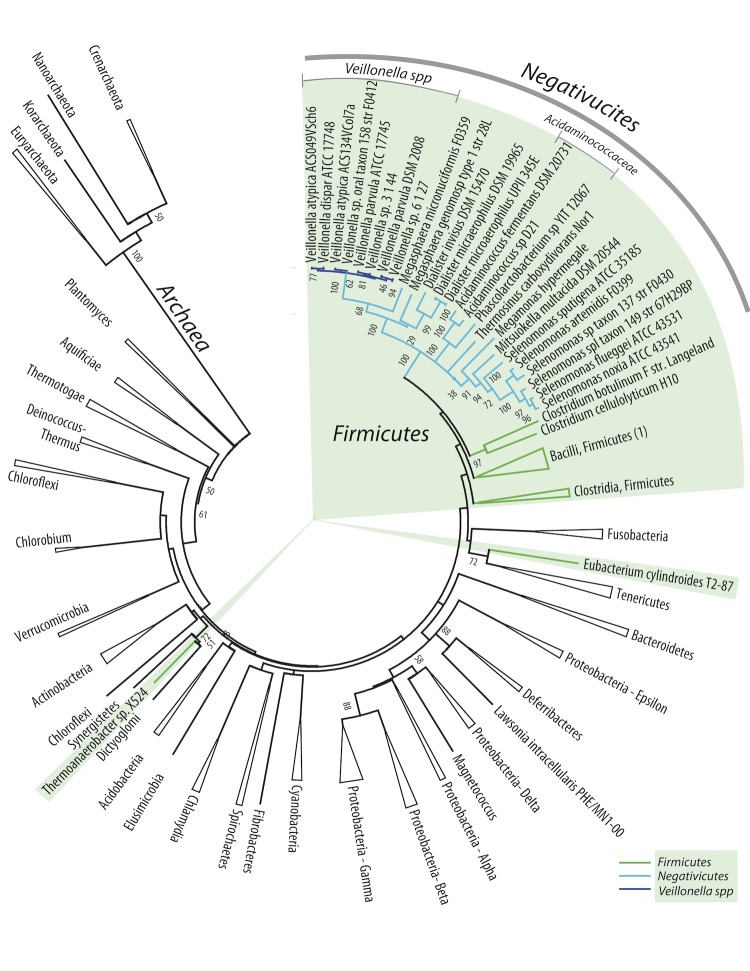
Phylogenetic neighbor-joining tree based on 16S rRNA genes extracted from 145 genomes (24 *Negativicutes* and 121 prokaryotic genomes representing 26 phyla). Bootstrap values of 50 and higher are indicated. With the exception of the *Negativicutes*, branches where all organisms belong to the same phyla are collapsed and named by the phyla they represent. The green shading indicates the position of *Firmicutes*. The collapsed branch of the *Bacilli,* marked (1), contains *Turicibacter sanguinis*, a *Firmicutes* member of the *Erysipelotrichales* as well as *Bacilli* members. An uncollapsed tree is included in the supplementary material.

Twenty-four *Negativicutes* genomes were compared to 121 other prokaryotic genomes covering 22 Bacterial and 4 Archaeal phyla. When available, at least two genomes were included for every phylum. The first analysis presented here is based on 16S rRNA alignments. A single 16S rRNA gene was extracted from each of the genomes and an alignment was produced spanning the maximum length of the gene. A phylogenetic tree was constructed based on this alignment, as shown in Figure 1. With the exception of the *Negativicutes*, branches of the tree were collapsed in those cases where the analyzed species within a phylum clustered together. With the exception of some *Firmicutes*, the analyzed genomes cluster according to their phylum, although the *Deferribacteres* phylum is mixed with the *Proteobacteria* phyla, and two members of *Proteobacteria* are not positioned with other members of their phylum (*Lawsonia intracellularis* and *Magnetococcus*). That most phyla could be collapsed is consistent with the weight of 16S rRNA similarities in currently accepted taxonomic descriptions of prokaryotes. The *Firmicutes*, however, show less consistency. Although most of the analyzed *Firmicutes* cluster together, two species are separated from the *Firmicutes* branch (*Eubacterium cylindroides* and *Thermoanaerobacter* sp., both members of *Clostridia*). The *Negativicutes* are positioned within the *Firmicutes* cluster, and this part of the tree is expanded in the figure for clarity. As can be seen, phylogeny of the 16S rRNA gene provides good resolution between the different genera of the analyzed *Negativicutes*. All *Veillonella* spp. are clustered within one branch of the *Negativicutes*. The *Acidaminococcaceae* (to which *Phascolarctobacterium* spp. also belong) are placed within the cluster of the *Veillonellaceae*, in accordance with their current classification [27]. The *Acidaminococcaceae* used to be recognized as a separate family within the *Negativicutes*, just like the *Veillonellaceae*, and during preparation of this contribution these two families were presented as such in the Taxonomy database at NCBI. Of note is the relatively close relationship between *Negativicutes* and two *Clostridium* species (*C. botulinum* and *C. cellulolyticum*), which does not cluster with other members of the *Clostridium* genus (Figure 1). That genus displays a high degree of variation and re-classification of some of the members of this genus is in progress (see for example [27]). That two members of the *Clostridia* are even placed outside the *Firmicutes* phylum is an indication of 16S rRNA gene sequence heterogeneity within this class.

**Figure 2:**
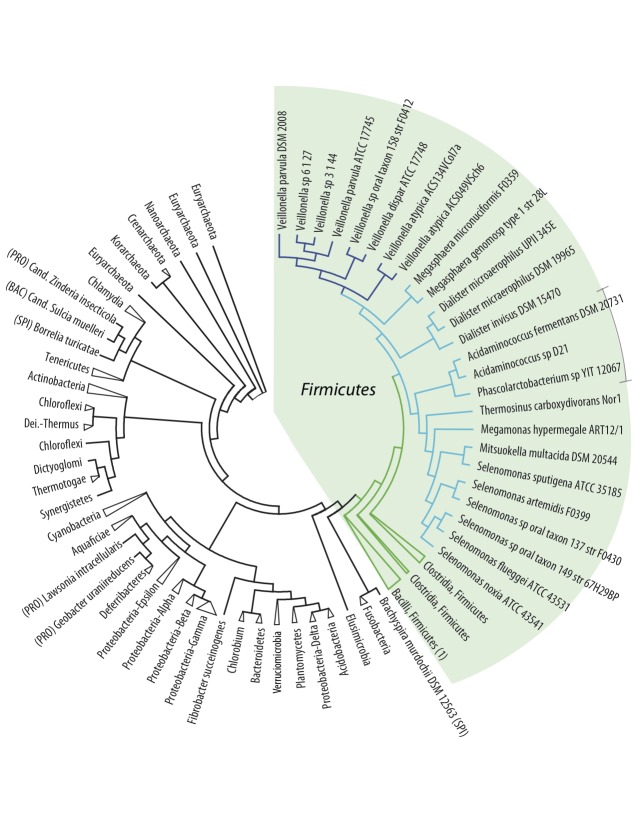
Phylogenetic tree based on composition vector analysis (CVtree) of all protein coding genes (amino acid sequences) derived from the analyzed genomes. Note that the branch lengths in this plot are artificial. The coloring is the same as in Figure 1 and branches have been collapsed. The *Firmicutes* branch *Bacilli*, marked (1), contains *Turicibacter sanguinis.* An uncollapsed tree is included in the supplementary material.

Next, all protein-coding genes of the analyzed genomes were compared and a composition vector tree (CVtree) was produced, based on amino acid sequences (Figure 2). The topology of the resulting tree is generally in accordance with the 16S rRNA tree shown in the previous figure. As indicated by the collapsed branches, the CVtree grouped most genomes according to their known taxonomic phyla, although not all *Spirochaetes* cluster together. In contrast to the 16S rRNA tree, in this protein tree all the *Firmicutes* cluster together, and are distinct from other phyla. The *Negativicutes* genomes, nested within the *Firmicutes*, again have the *Acidaminococcaceae* placed within the *Veillonellaceae*, while all *Veillonella* spp. are found in one cluster. All *Clostridia*, this time divided into two collapsed branches, are positioned as the closest relatives to *Negativicutes*. It is of interest that among the closest relatives to *Firmicutes*, based on this analysis, are the *Fusobacteria* and the *Elusimicrobia*; these are atypical diderm bacteria that produce lipopolysaccharides [28]. However, the spirochete*, Brachyspira murdochii,* does not possess two membranes, but is nevertheless grouped with atypical diderms. On the other hand while the *Synergistetes* are atypical diderm bacteria, they are placed elsewhere in the tree (Figure 2).

**Figure 3:**
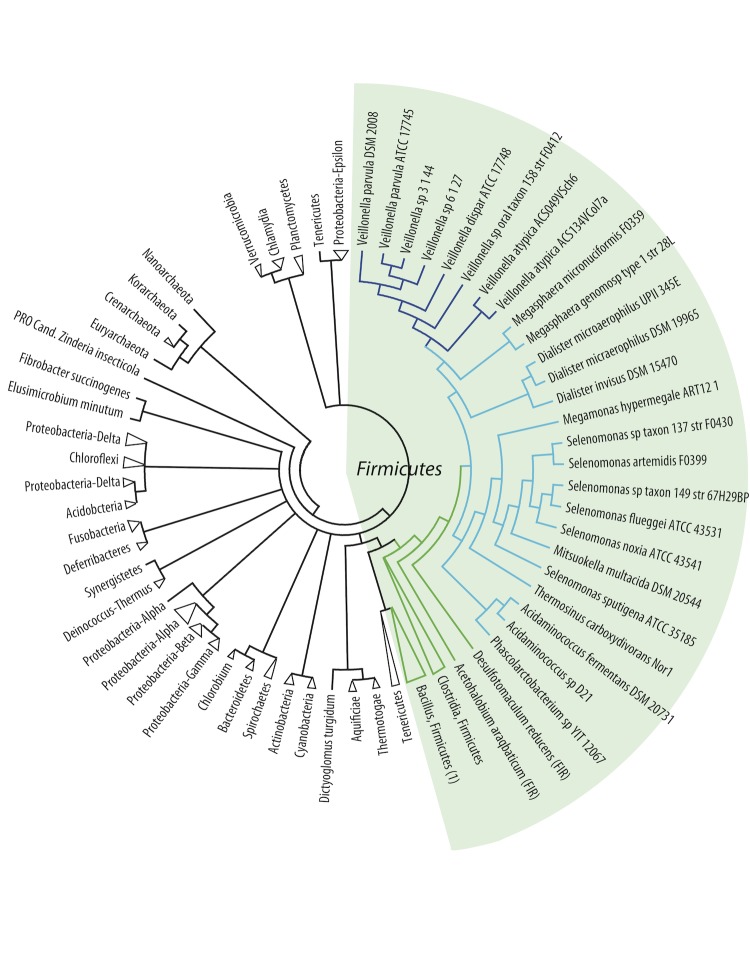
Consensus tree based on the phylogenetic trees of 27 genes conserved in all 145 genomes. The collapsed branch of the *Bacilli,* marked (1), contains *Turicibacter sanguinis.* An uncollapsed tree is available as a supplemental figure.

A third analysis was based on a subset of proteins found conserved amongst all analyzed genomes. These conserved proteins were selected based on a protein BLAST (a cutoff of 50% identity and 50% coverage of the query length was used) and single linkage clustering. The analysis identified 29 genes that are shared among all 145 genomes [Table 2]. A consensus tree was constructed based on these 29 conserved proteins (Figure 3). The results confirm the global observations of the other two phylogenetic analyses: the *Negativicutes* cluster together and are most closely related to *Clostridia* (in this case the most closely related species are *Desulfotomaculum reducens* and *Acetohalobium arabaticum*). As before, the *Acidaminococcaceae* cluster together but within the *Veillonellaceae*. The position of *Turicibacter sanguinis* within the *Bacilli* group of *Firmicutes* is consistent with the other two trees but contrasts with its taxonomic description at NCBI as a member of the *Erysipelotrichia*.

**Table 2:** Universally conserved COGs

Group	Average length (aa)	Annotation
COG0012	380	Predicted GTPase, probable translation factor
COG0016	423	Phenylalanine-tRNA synthethase alpha subunit
COG0048	137	Ribosomal protein S12
COG0049	182	Ribosomal protein S7
COG0052	240	Ribosomal protein S2
COG0080	154	Ribosomal protein L11
COG0081	230	Ribosomal protein L1
COG0087	288	Ribosomal protein L3
COG0091	157	Ribosomal protein L22
COG0092	240	Ribosomal protein S3
COG0093	130	Ribosomal protein L14
COG0094	182	Ribosomal protein L5
COG0096	131	Ribosomal protein S8
COG0097	177	Ribosomal protein L6P/L9E
COG0098	220	Ribosomal protein S5
COG0100	145	Ribosomal protein S11
COG0102	167	Ribosomal protein L13
COG0103	172	Ribosomal protein S9
COG0172	442	Seryl-tRNA synthetase
COG0184	154	Ribosomal protein S15P/S13E
COG0186	122	Ribosomal protein S17
COG0197	175	Ribosomal protein L16/L10E
COG0200	166	Ribosomal protein L15
COG0201	445	Preprotein translocase subunit SecY
COG0202	323	DNA-directed RNA polymerase, alpha subunit
COG0256	178	Ribosomal protein L18
COG0495	854	Leucyl-tRNA synthetase
COG0522	199	Ribosomal protein S4 and related proteins
COG0533	375	Metal-dependent proteases with chaperone activity

In conclusion, based on three independent phylogenetic analyses, the closest relatives to the *Negativicutes* seem to be the *Clostridiaceae*. The observed clustering of species within the *Negativicutes* is consistent with their assigned taxonomy. Furthermore, these analyses show that *Veillonella spp.* form a distinct branch, most different from the other *Negativicutes*, while the recent change of status of the *Acidaminococcaceae* (they are no longer a separate family) is confirmed by these analyses.

Apart from comparing proteins and genes, genomes can also be compared based on nucleotide composition irrespective of their coding capacity. For instance, the frequency of nucleotide combinations can reveal similarities between genomes that are independent of protein-coding information. We compared the frequency of tetranucleotides for all 145 genomes. The observed frequency of all 64 tetranucleotide combinations was extracted for each genome and these frequencies were divided by the theoretically calculated, expected frequencies (corrected for differences in base composition). This ratio, which could be interpreted as a genomic signature, was expected to reflect taxonomic divisions [29]. However, although the analysis identified a high similarity in tetranucleotide frequency for all of the analyzed *Veillonella* genomes, most of the clustering observed was not in accordance with known taxonomic relationships. Not only were *Negativicutes* other than *Veillonella* separated from each other and strewn across the phyla, but also several other *Firmicutes* were distributed over various branches (data shown as supplementary material). In fact, for most of the analyzed genomes, members of identical phyla did not cluster together and even the *Archaea* were mixed with *Bacteria*, although some closely related species were indeed clustered. This may explain why all *Veillonella* genomes grouped together. Several organisms with similar tetranucleotide frequencies did not share a common ecological niche, in contrast to previously reported observations (reviewed in [30]). Neither was the obtained clustering dictated by GC-content. The conclusion from this analysis was that tetranucleotide analysis is only taxonomically informative for closely related genomes.

**Figure 4:**
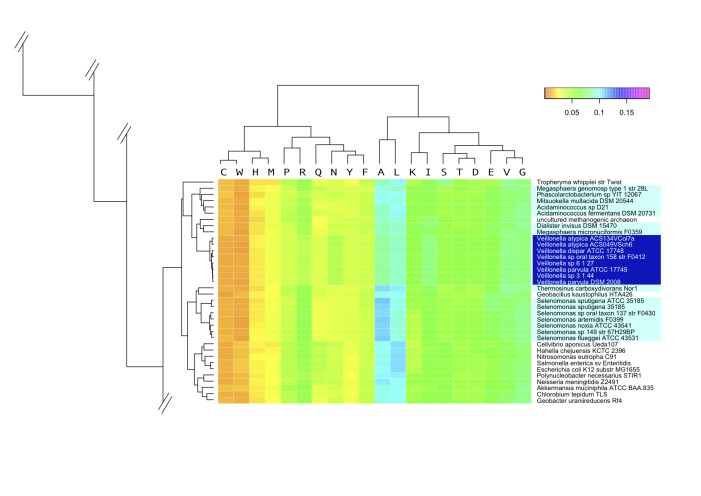
A zoomed heatmap of the amino acid frequency found in the deduced proteomes of all 145 genomes. A fragment of the heatmap is shown, presenting the cluster in which all but two *Negativicutes* are found. The remaining two, both *Dialister microaerophilus* genomes, are positioned elsewhere in the tree, closest to *Clostridium cellulolyticum* (not shown in this zoom). The color scale indicates highly underrepresented (orange) to highly overrepresented amino acid frequency (magentum). The full figure is available as supplementary information.

We also compared whole-genome amino acid frequencies in each of the deduced proteomes. Although the results are slightly more in agreement with known taxonomy as compared with the genomic signatures discussed above, this analysis does not cluster organisms according to their phyla, and again some *Archaea* are mixed with *Bacteria*. The relevant part of the heatmap based on amino acid frequency is shown in Figure 4. All *Veillonella* genomes cluster together within the *Negativicutes*, with the exception of two of the three *Dialister* genomes, which are found most closely related to *Clostridium* species (See supplemental information for a version of this figure showing all the genomes). The major *Negativicutes* cluster also contains a *Geobacillus* (which is a Gram-positive *Firmicutes*) and a methanogenic Archaean. Interestingly, the closest relatives to this cluster are not *Clostridia*, as the previous phylogenetic trees suggest, but a number of *Proteobacteria*. It is striking that the amino acid frequency analysis detects similarities to *Proteobacteria*, with which the *Negativicutes* have their two membranes in common.

**Figure 5:**
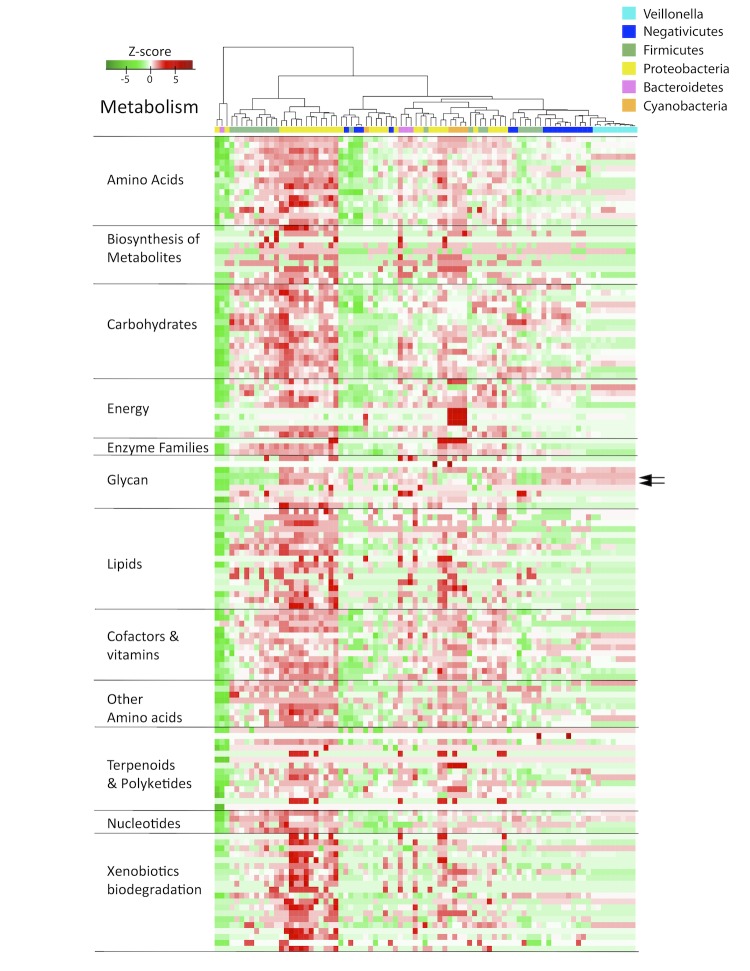
Heatmap of metabolism potential, based on Kyoto Encyclopedia of Genes and Genomes ontology (KEGG). The green color in the heatmap indicates weak metabolic potential, while red signals strong potential. The arrows to the right indicate the scores for lipopolysaccharide biosynthesis. A version summarizing the metabolism pathways and showing the species legend is available as supplementary material.

The metabolic properties encoded by the genomes were analyzed next, based on KEGG comparisons [24]. The results are again visualized in a heatmap (Figure 5). We hypothesized that this analysis could identify similarities based on niche adaptation. For simplicity, only a selected number of phyla are shown: apart from the *Firmicutes*, genomes are included that represent *Bacteroidetes* and *Proteobacteria* (both of which contain members frequently found in the oral or gut microbiome), while *Cyanobacteria* are included as representatives of a phylum that occupy an environmental niche. Since the genomes are compared based on predicted proteomes, their annotation was standardized in order to reduce artificial variation caused by gene annotation differences. As can be seen in Figure 5, the *Veillonella* genomes all cluster together at the right-hand side of the plot, within a larger cluster containing most of the other *Negativicutes* and some *Firmicutes*. The three *Dialister* species are placed outside the *Negativicutes* cluster. The other *Firmicutes* that are found combined with the *Negativicutes*, based on their metabolic potential, are *Clostridium cellulolyticum, Eubacterium rectale, Lactococcus lactis, Streptococcus pneumoniae* and *Turicibacter sanguinis*. These are all common members of the oral or intestine microbiome. As expected, the metabolic pathway for lipopolysaccharide biosynthesis is shared between the *Negativicutes* and other Gram-negative species, as indicated by the arrows in Figure 5. Interestingly, the *Cyanobacteria* form a small cluster within, not outside the tree, together with a *Haliangium* and a *Sorangium* species as their closest neighbors (both are social *Myxococcales* belonging to the *Deltaproteobacteria*). The exclusive ability of carbon fixation by *Cyanobacteria* is apparent from the dark red square in the block 'energy'. The lanes of *Veillonella* in Figure 5 are dominated by light colors, indicative of medium metabolic potential; that is, in contrast to some genomes where most of the pathways are present (dark red for Proteobacteria for example) or missing (dark green for other *Negativicutes*), the *Veillonella* genomes have partial pathways (based on knowledge primarily from aerobic genomes). There is no reason to believe that the *Veillonella* genomes should have less metabolic potential than other *Negativicutes*. Indeed, it is likely that the differences in metabolic potential of *Veillonella* are truly reflective of alternative capabilities for these bacteria.

**Figure 6:**
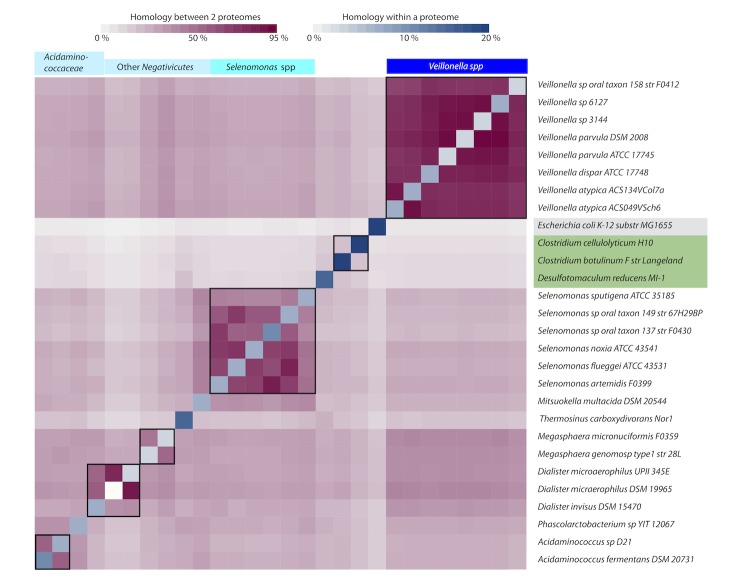
Proteome comparison represented by a BLAST matrix, based on 24 *Negativicutes* genomes with reciprocal best hits. The genomes of *Clostridium botulinum*, *Cl. cellulolyticum*, *Desulfotomaculum reducens* and *E. coli* are added for comparison. Inter-genus comparisons are indicated by black squares. A version reporting the numerical values of homology percentages is available as supplementary information.

It was further investigated how conserved the predicted proteomes are within the *Negativicutes*. As a quantitative measure for homology, shared protein-coding genes were identified by pairwise BLASTP comparison and expressed as a percentage of the combined proteomes. The results are shown in a matrix (Figure 6). In addition to the proteomes of the 24 *Negativicutes*, the comparison includes *Clostridium botulinum*, *Cl. cellulolyticum* and *Desulfotomaculum reducens*, as these *Firmicutes* were shown to share characteristics with *Negativicutes* in previous analyses (*cf*. Figures 1 and 3). The proteome of *E. coli* K12 is included as an example of a Gram-negative intestinal bacterium. The BLAST matrix was constructed using reciprocal best BLAST hits to determine the presence of shared protein family between two genomes. Inspection of Figure 6 shows that the genus *Veillonella* is relatively homogeneous; any two members of this genus share between 67% and 90% homology (1,357 to 1,682 protein families), irrespective of the species. The genus *Selenomonas* is more heterogeneous, with pairwise homology varying from 42% to 82% between any two species (980 to 1659 protein families). The three proteomes of *Dialister spp.*, covering two species, share between 40% and 84% homology. The highest homologous fraction identified between two members of different genera within the *Negativicutes* is 43% (*Mitsuokella multacida* compared to *Selenomonas sputigena*, whereas the lowest homology is 15% (*Dialister* spp. compared to *Thermosinus carboxydivorans*). *Negativicutes* share between 9% and 33% homology with the analyzed *Firmicutes*, whereas slightly lower homology is detected with *E. coli* (between 7% and 24%).

Finally, we assessed the gene pool conserved within all analyzed *Negativicutes*. Using the same cutoff for protein BLAST comparison as before, a core-genome is identified that contains about 300 conserved protein families (data not shown). This is a relatively low number of conserved proteins, reflective of the extensive genetic heterogeneity within this bacterial class.

## Discussion

The availability of complete sequences for a large and diverse set of Bacterial genomes has helped in exploring the conundrum of the genus *Veillonella*, a genus within the *Negativicutes* class, all of which are Gram negative *Firmicutes*. The 16S rRNA tree shown as Figure 1 illustrates how “close” the *Negativicutes* are to other *Firmicutes*. The closest Gram positive *Clostridium* species are actually quite distant to *Veillonella* and other *Negativicutes* genomes, as can be seen in the low fraction of shared protein families in Figure 6. The Gram-negative *Firmicutes* are even more distant to other Gram negatives, such as *Proteobacteria* (e.g., *E. coli*). It should be noted that the family *Clostridiaceae* is a largely diverse group with many members being re-classified [27]. It is therefore possible that the taxonomic description of some *Clostridium* genomes may change in future. However, our analyses did not identify one single Gram-positive *Firmicutes* (*Clostrida* or others) that consistently was identified as most closely related to *Veillonella*. As seen from three types of phylogenetic analysis, the *Negativicutes* class genomes form a distinct cluster within the *Firmicutes*, and the *Veillonella* genus forms a relatively homogeneous group of species within the *Negativicutes*, with relatively conserved metabolic properties (Figure 5). In comparison, the *Selenomonas* genus is more heterogeneous, at least based on their total gene comparison, as illustrated in Figure 6.

In contrast to expectations, relatively little homology between *Negativicutes* and other Gram-negative genomes was detected in our analyses. Neither gene-dependent phylogenetic analysis, nor gene-independent DNA tetramer analysis identified a significant commonness between *Negativicutes* and, say, *Proteobacteria*. Only whole-genome frequency analysis of amino acid usage identified some similarity to a few *Proteobacteria*, and this might be more reflective of environment the organism is adapted to, and not phylogeny. Using KEGG pathways for metabolic comparison of the proteomes we found few pathways in common, with the exception of a shared lipopolysaccharide biosynthesis pathway. From all analyses combined, it is clear that the taxonomic placement of *Negativicutes* within the *Firmicutes* reflects their genetic and genomic characteristics, although the proteins encoded by the *Negativicutes* genomes are quite distinct from their Gram-positive cousins. It could be speculated that the double membrane of the *Negativicutes* evolved in a lineage that used to be a single-membrane (Gram-positive) Firmicute. Whether this event co-evolved independently of the formation of other Gram-negative phyla, or was the result of lateral gene transfer, cannot be stated for certain at present; estimations of horizontally transferred regions in *Veillonella parvula* DSM 2008, the only fully assembled *Veillonella* genome available, using the least conservative method on the Islandviewer web-site [31], revealed that only 2% of the genome is of foreign origin. In comparison, 9% of the *E. coli* K-12 subsp. MG1655 genome was predicted as horizontally transferred. Further analyses are therefore needed to assess this in more detail.

### Author’s contributions

Tammi Vesth was a main contributor to the writing of the manuscript and to the organization of the work. Trudy Wassenaar helped considerably in editing and improving the manuscript. Individual contributions: Asli Ozen (16s rRNA and CV tree), Oksana Lukjancenko (consensus tree), Sandra Andersen (initial investigations and background research, early version of the manuscript), Rolf Sommer Kaas (BLAST matrix), Jon Bohlin (tetramer and amino acid usage heatmaps), Intawat Nookaew (metabolism heatmaps). David Ussery provided the original idea for this manuscript, suggested the figures, helped in early drafts of the manuscript, and supervised the project.
